# Leprosy in children in Cuba: Epidemiological and clinical description of 50 cases from 2012–2019

**DOI:** 10.1371/journal.pntd.0009910

**Published:** 2021-10-28

**Authors:** Raisa Rumbaut Castillo, Laura C. Hurtado Gascón, Jenny Laura Ruiz-Fuentes, Fernanda M. Pastrana Fundora, César R. Ramírez Albajés, Andres F. Henao-Martínez, Carlos Franco-Paredes, Ángel Arturo Escobedo

**Affiliations:** 1 Ministerio de Salud Pública, La Habana, Cuba; 2 Centro Provincial de Higiene y Epidemiología, La Habana, Cuba; 3 Laboratorio Nacional de Referencia e Investigaciones en Tuberculosis, Lepra y otras Micobacterias, Instituto de Medicina Tropical Pedro Kourí, La Habana, Cuba; 4 Hospital Juan Manuel Marqués, La Habana, Cuba; 5 Facultad de Ciencias Médicas Finlay- Albarrán, La Habana, Cuba; 6 University of Colorado, Anschutz Medical Center, Division of Infectious Diseases, Aurora, Colorado, United States of America; 7 Hospital Infantil de México, Federico Gómez, México City, México; 8 Instituto de Gastroenterología, La Habana, Cuba; Shandong Provincial Institute of Dermatology and Venereology, CHINA

## Abstract

**Introduction:**

In 1993, Cuba achieved leprosy elimination according to the World Health Organization’s (WHO) indicator of less than one case per 10,000 population. Despite this achievement, detection of new cases occurs every year among all age groups including children. Detection of new cases in children reveals persistent transmission of the infection.

**Objective:**

To describe the clinical and epidemiological features of leprosy in individuals younger than 15 years (childhood leprosy) reported to the Cuban National Leprosy Control Program (NLCP) between 2012 and 2019.

**Methods:**

We conducted a retrospective descriptive study between 2012 and 2019 to assess the clinical and epidemiologic features of individuals under the age of 15 years with a confirmed diagnosis of leprosy reported to the NLCP. We reviewed the NLCP database and collected data to better define the total number of cases of leprosy in adults, children (younger than 15 years). We assessed socio-demographic variables (age, gender, and province of residence) as well as variables of clinical interest including operational classification and staging at diagnosis, bacillary index, grade of disability by WHO staging. Additionally, we evaluated epidemiological variables including passive versus active surveillance of cases, contact investigation focusing specifically in household transmission, and the degree of kinship as well as standing of the child within the focus of transmission when there were additional cases.

**Results:**

We identified fifty children during the study period corresponding to 3% of the overall cases of leprosy comprising all age groups in Cuba. In the age group younger than 15 years, the majorities of cases was from the Granma province and most were between the ages of 10 and 14 years. Clinically, multibacillary/lepromatous forms were the most common type identified with positive bacillary index. The majority of children diagnosed with leprosy during our study period had a history of a relative with a confirmed diagnosis of leprosy.

**Conclusions:**

Detection of cases of leprosy in individuals younger than 15 years of age in Cuba demonstrates ongoing transmission of *M*. *leprae* in specific geographic hotspots. Its frequency in the early adolescence, the predominant clinical forms, and the mode of detection associated with sources of suspected familiar infection demonstrated that there is a need for further efforts by the NLCP to conduct active surveillance activities among affected communities to identify cases of leprosy earlier with the goal of preventing further household and community transmission.

## Author summary

Leprosy is a chronic mycobacterial infection that affects the skin and peripheral nerves. Despite the wide deployment of effective anti-mycobacterial therapy for this infection, there are still approximately 200,000 new cases of leprosy identified every year globally. The WHO leprosy elimination strategy reduced the overall prevalence of the disease, but the detection of new cases indicates ongoing transmission in many countries. The detection of cases of leprosy in children is a surrogate marker of ongoing transmission in the community. In this study conducted in Cuba, we identified that the Cuban National Leprosy Control Program (NLCP) continues to see an important number of cases of leprosy in children (under the age of 15 years) in selected communities in many of the Cuban provinces. Additionally, we observed that in most cases either one or more relatives with leprosy residing in the same household. In summary, our study highlights to achieve interrupting of leprosy transmission in Cuba; there is a need for further active surveillance efforts targeting selected provinces and communities to identify cases early in their clinical course and institute antimicrobial therapy. Contact investigation is one of the main tools to achieve interruption of transmission.

## Introduction

Leprosy is a chronic mycobacterial infection caused by *Mycobacterium leprae* or *Mycobacterium lepromatosis* [1]. The disease involves the skin, mucosae, the upper respiratory tract, eyes, and peripheral nerves, as the target cells of the infection include histiocytes and Schwann cells. Multibacillary forms of the disease affect several organs and systems, making the disease complex and systemic [2]. The disease often produces irreversible nerve damage, which manifests as severe neurologic dysfunction, deformity, and disability. Additionally, the disease causes substantial stigma and social isolation due to skin lesions, deformities, and limb loss [3,4]. Globally, most leprosy cases are identified in adults, but a significant number of cases are also identified in individuals younger than 15 years [5]. There are environmental as well as genetic predisposition factors that contribute to the clinical spectrum and differential expression of the polar and borderline forms of leprosy [6].

The World Health Organization (WHO) continues to report approximately 200,000 new cases of leprosy every year. There have been more than 4 million new cases reported to WHO, since the elimination of leprosy as a public health threat by WHO in the year 2000. Among these cases, many occur in children. For example, in 2018, among the 208,619 new cases reported by WHO, 16,013 were diagnosed in children. Of these children, grade II disability was present at the time of diagnosis in more than 350 of them [7–9]. The persistent occurrence of leprosy in the pediatric age (children younger than 15 years) correlates with active transmission in the community [10–12]. Furthermore, the continuing identification of grade II disability (loss of protective sensation damage or visible deformity in hands and/or feet) in children at the time of their diagnosis reflects an unsatisfactory strategy to interrupt leprosy transmission and highlights delays in timely detection of cases.

In 1962, the Cuban government established the Cuban National Leprosy Control Program (NLCP). Since its inception, this program has abided to the international recommendations for the management and control of the disease [13]. As a result of the activities of the NLCP, Cuba achieved the national elimination of leprosy as a public health concern by 1993 according to the WHO indicator of a prevalence rate of less than one case per 10,000 inhabitants [13,14]. All provinces in Cuba shared this same achievement equally by 2003. Despite this achievement, every year there are many reports to the NLCP of leprosy in children [15].

Cuba is classified by WHO as a country in the post-elimination phase of leprosy. However, there are some gaps in leprosy control efforts to fulfill the mission of the NLCP, hence, the interruption of leprosy transmission has not occurred. Achievement of this noble goal requires reassessing surveillance and control actions in accordance to the goals of the WHO global leprosy strategy 2016–2020 to accelerate actions towards a world without leprosy [16]. Recently, we carried out a study about the Cuban experience in leprosy control activities from 2000 to 2017, in which socio-demographic and epidemiological variables were included along with serology and PCR testing to implement interventions for the early diagnosis of cases of leprosy during childhood [17]. Since childhood leprosy is an epidemiological indicator of persistent community transmission, we were interested in assessing all reported cases of leprosy in children in Cuba from 2012–2019, including new variables and considering a wider epidemiological approach. Our main objective was to identify possible missed opportunities or gaps in surveillance activities that could contribute to implement interventions, such as active surveillance with thorough investigations of contacts.

## Methods

### Ethics statement

The study was approved by the ethics committee of the National Technical Leprosy Advisory Commission.

### Study description

We conducted a descriptive retrospective study to identify the clinical and epidemiological features of leprosy cases in individuals under the age of 15 years diagnosed in Cuba between January 2012 and December 2019. We obtained demographic, clinical, and epidemiological information from the NLCP database for each of the cases of leprosy in childhood. This database is updated annually with the information collected from case reports notifications and through conducting epidemiological surveys, as well as summaries of the clinical history of each reported case.

We analyzed the following variables: a) Number of cases of leprosy in all age groups: includes the total of all new cases detected in the country per year in the study period and the accumulated ones for the same period; b) Number of childhood cases of leprosy(children under 15 years of age): includes the total of all children detected in the country by year in the study period and the accumulated of the same period; c) Overall detection rate and detection rate in children per 100,000 inhabitants per year; and d) Proportion of leprosy cases in childhood: number of new cases of leprosy for each year during the study period, and the accumulated number of the same period.

Among the socio-demographic variables we assessed: a) Age ranges (0–4 years) (5–9 years) and (10–14 years); b) gender, and c) Province of residence at the time of leprosy diagnosis. We selected the following clinical variables: a) Operational classification: multibacillary and paucibacillary according to the 1998 WHO staging [18]; b) Bacillary index at the time of diagnosis and referring to the results of the smear microscopy and with a range that oscillates between 0 and 6, according to a logarithmic scale [19,20]; and c) Degree of disability at diagnosis: identified in a range from 0 to 2, according to the WHO classification of degree of disability [21].

Finally, we analyzed surveillance and contact investigation data using the following variables: a) passive case detection (those patients who come spontaneously for consultation) versus active surveillance (cases detected during active screening carried out by health personnel during contact investigation or through surveillance activities conducted in high-risk areas); b) Timing of diagnosis: early, when the time between the date of onset of symptoms (as referred by the patient)and the diagnosis is less than 12 months; and late when it is 12 months and more; c) Potential source of infection: suspected or not suspected according to the results of the epidemiological investigation among people with a past or present diagnosis of leprosy in close contact with the child and who are not part of the family nucleus; d) Family history of leprosy: presence or absence of a relative(parents, siblings, grandparents and other relatives) with leprosy or history of treated leprosy; e) Degree of kinship with the child: referring to relatives with leprosy or with a history of leprosy and their kinship with the minor; e) Position of the child within the focus of transmission: single-case, two secondary cases, three secondary cases or more than four cases within the same focus of transmission.

### Analysis

We performed univariate analysis. We tabulated results in Microsoft Excel 2016 tables. We reported summary measures with absolute numbers, relative numbers, and rates.

## Results

The NLCP received reports of 1,689 new cases of leprosy during the study period. There were 50 cases reported in individuals younger than 15 years corresponding to 3.0% of all new diagnoses during the study period. Detection rates in children ranged from 0.03 to 0.10 per 100,000 inhabitants, and the proportion of cases in this age group did not exceed 4.7% (Table 1). There were 30 cases (60%) in the age range of 10 to 14 years, and the report of youngest child during the study period was in a three years old child (Table 2). Male sex predominated with 26 cases (52.0%). Granma province contributed with the highest number of diagnoses, 18 (36.0%); followed by Ciego de Ávila and La Habana with 7 cases each (14%) (Table 2) and this was the same order in the proportion of cases of childhood leprosy in relation to new cases (Table 3).

**Table 1:** Detection of leprosy cases in adults and children in Cuba 2012–2019.

Year	Cases	Rate*	Cases in Children	Rate *	%
**2012**	258	2.3	9	0.08	3.5
**2013**	232	2.1	11	0.10	4.7
**2014**	210	1.9	8	0.07	3.8
**2015**	207	1.8	9	0.08	4.3
**2016**	186	1.7	3	0.03	1.6
**2017**	190	1.7	5	0.04	2.6
**2018**	220	2.0	3	0.03	1.4
**2019**	186	1.7	2	0.02	1.1
**Total**	**1689**		**50**		**3.0**

*per 100 000 population

**Table 2:** Demographics of childhood leprosy (N = 50)*.

Variables	Features	Number of children cases	%
**Age**	0–4	2	4.0
	5–9	18	36.0
	10–14	30	60.0
**Gender**	Male	26	52.0
	Female	24	48.0
**Province**	Granma	18	36.0
	Ciego de Ávila	7	14.0
	La Habana	7	14.0
	Guantánamo	5	10.0
	Holguín	4	8.0
	Las Tunas	2	4.0
	Santiago de Cuba	2	4.0
	Pinar de Rio	1	2.0
	Matanzas	1	2.0
	Cienfuegos	1	2.0
	Villa Clara	1	2.0
	Camaguey	1	2.0

*Number of cases of childhood leprosy

**Table 3:** Detection of leprosy cases in adults and children by province. Cuba 2012–2019.

Province	Total of leprosy cases	Number of children cases	%
Granma	346	18	5.2
Ciego de Ávila	144	7	4.9
La Habana	146	7	4.8
Guantánamo	189	5	2.6
Holguín	96	4	4.2
Las Tunas	43	2	4.7
Santiago de Cuba	244	2	0.8
Pinar de Rio	53	1	1.9
Matanzas	38	1	2.6
Cienfuegos	35	1	2.9
Villa Clara	61	1	1.6
Camaguey	151	1	0.7

**Fig 1:**
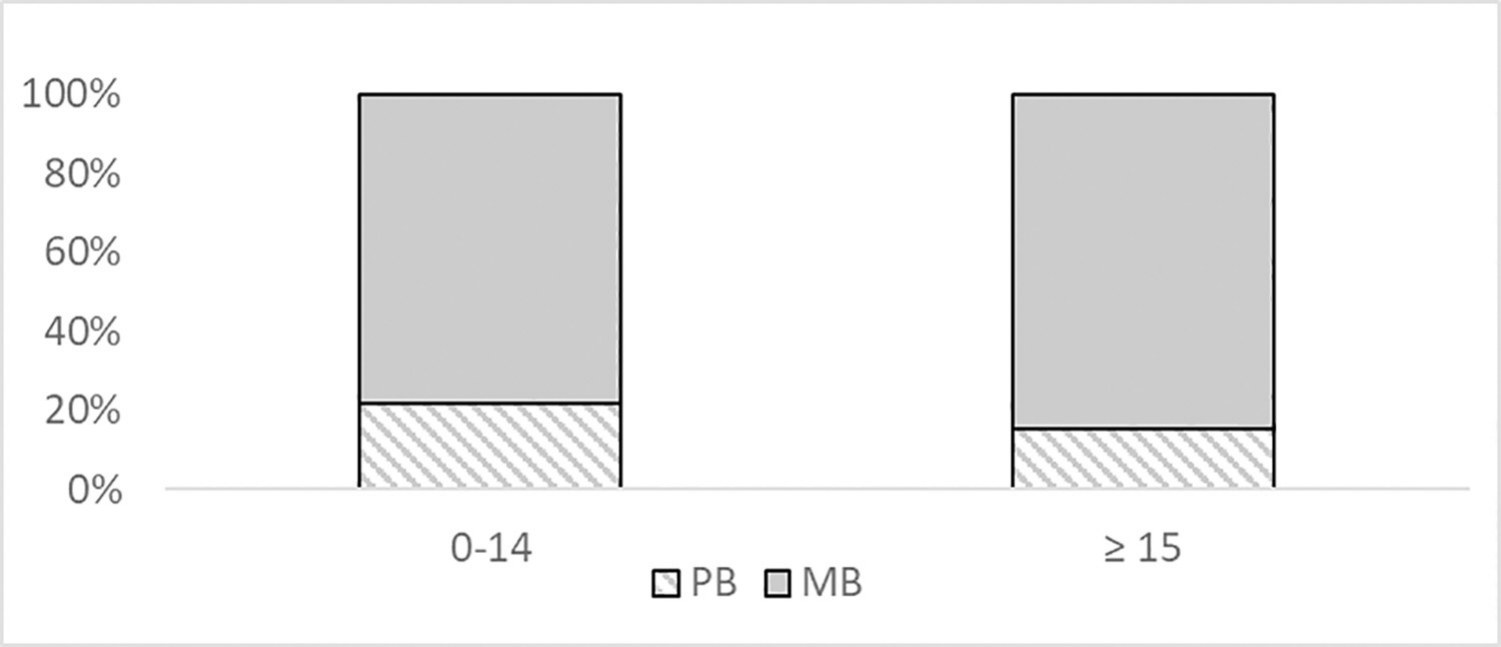
Multibacillary and paucibacillary case of leprosy by age groups in Cuba, 2012–2019.

Almost 80% of the pediatric cases of leprosy were identified as multibacillary [39/50 (78.0%)]. **Fig 1** shows a similar pattern in the adult population. It was observed that 27 of the 50 patients had positive skin smears, and only two children reported disability at the time of their diagnosis (Table 4).

**Table 4:** Clinical features of childhood leprosy in Cuba (n = 50).

Variable	Clinical Features	Number of children cases	%
**Leprosy subtype**	Multibacillary	39	78.0
	Paucibacillary	11	22.0
**Bacillary index at the time of diagnosis**	0	23	46.0
	1	17	34.0
	2	6	12.0
	3	2	4.0
	4	1	2.0
	5	1	2.0
**Grade of disability**	0	48	96.0
	1	1	2.0
	2	1	2.0

Most reports of children with confirmed leprosy occurred early in the natural history of the infection through passive surveillance (Table 5). Detection of cases by active surveillance was associated with early age at diagnosis, as well as the timeliness of diagnosis, and to negativity of the smear microscopy. However, in both forms of surveillance, there were mostly children with multibacillary forms of the disease (Table 6).

**Table 5:** Childhood leprosy cases according to surveillance and epidemiological screening (N = 50).

Variable	Features	Number of children cases	%
**Detection mode**	Passive surveillance	30	60.0
	Active surveillance in -high-risk areas	2	4.0
	-close contact	18	36.0
**Timing of diagnosis**	Early	45	90.0
	Late	5	10.0
**Suspected potential source of Infection**	Yes	29	58.0
	No	21	42.0
**History of leprosy in a family member**	Yes	27	54.0
	No	23	46.0
**Relative with confirmed diagnosis of leprosy (N = 27)** *	Mother	8	29.6
	Father	5	18.5
	Grandparent	10	37.0
	Sibling	4	14.8

* Children with family history of leprosy

**Table 6:** Distribution of some variables according to the surveillance method.

	Number of children cases by Passive surveillance (n = 30)		Number of children cases by Active surveillance (n = 20)	
Variables		%		%
**Age**				
0–4	1	3.3	1	5.0
5–9	8	26.6	10	50.0
10–14	21	70.0	9	45.0
**Leprosy subtype**				
Multibacillary	23	76.0	15	75.0
Paucibacillary	7	23.0	5	25.0
**Timing of diagnosis**				
Early	25	85.3	19	95.0
Late	5	16.6	1	5.0
**Bacillary index**				
Positive	19	63.3	9	45.0
Negative	11	36.6	11	55.0

All reported contacts were investigated for a history of leprosy or presence of active leprosy. We were able to identify the source case of infection in 29 cases. In most cases, we identified that there were concomitant cases of leprosy or history of leprosy in relatives in the same household. Grandparents were the next of kin with a history of leprosy or active leprosy associated with cases of pediatric leprosy. Additionally, when conducting an epidemiological investigation of the child’s position within the cluster of cases, 42% of the children were regarded as the only ‘one case identified’ of the specific focus of transmission (Table 7).

**Table 7:** Position of the child within the focus of transmission (n = 50).

Variable	Number of children cases by	%
**Only one case identified**	21	42.0
**Only secondary case identified**	15	30.0
**Secondary case with other three associated cases**	8	16.0
**Secondary case with other four associated cases**	4	8.0
**Secondary case with other five associated cases**	2	4.0

## Discussion

Annually, there are more than 200 new cases of leprosy identified in Cuba. Globally, Cuba is considered a low prevalence country, due to the low rates it maintains; however, in the Americas region, territories with more than 100 new cases per year are considered as countries with high burden for the disease [22]. Unfortunately, Cuba is therefore classified as a high burden of disease which is compounded by high number and new diagnoses of childhood leprosy. In 2020, other countries in Latin America were also categorized as having a high burden of leprosy transmission including Brazil, Argentina, Colombia, Mexico, Paraguay and Venezuela. Of the overall number of new cases of childhood leprosy reported in 2020, Cuba and Paraguay followed Brazil with the highest number of cases. [9].

While we continue to detect cases of leprosy in children in Cuba, our study shows that there has been a decrease in the proportion of children diagnosed with this infection in recent years, which may obey to a natural decline in transmission in the general population, or potentially as the result of policies implemented by the NLCP. However, despite Cuban health system invests substantial time, effort and financial resources on leprosy control, other factors could also have contributed towards the relative decline of new diagnosis during these years. These include: the focus of the scientific community on other ‘more fashionable’ diseases or pressing activities to the national health system during outbreaks (i.e., cholera, dengue, zika, chikungunya, international Ebola alert, and conjunctivitis). There are multiple factors that limit early case detection of cases of leprosy than range from missed opportunities for diagnosis in clinical settings due to its non-specifical clinical signs, its ability to masquerade other conditions, and the insufficient number of healthcare providers trained to identify and treat cases of leprosy. Due to the stigma associated with leprosy, many people do not reach the healthcare system. From a public health perspective, other public health priorities such as the COVID-19 pandemic pose a threat to the sustainability of surveillance and control efforts.

Nevertheless, the persistent identification of cases of leprosy in children is an indicator of ongoing and recent transmission [23]. Certainly, from a numerical point of view the cases are few and represent low detection rates, a single case of leprosy in individuals younger than 15 years of age is considered as too many. Leprosy is slow to develop due to its incubation period (5 to 7 years on average) and therefore it is an indirect marker of recent transmission [24]. Huggi asserts that leprosy in children is more frequent between 5–14 years of age, which coincides with findings from other authors such as Freitas de Corpes and Balai, who in their studies found 66.1% and 80%, respectively, in the 10 to 14-year-old age group in children from Brazil and India [25–27]. In the current study, we found a similar trend of leprosy to occur predominantly in the 10–14 age group. Interestingly, we also identified a case in a 3-year old child supporting the presence of ongoing transmission of leprosy in some communities in Cuba. However, transmission in child younger than 1 year of age appears to occur only in highly endemic territories [28].

The proportion of cases of pediatric leprosy by gender was not significantly different, with a slight predominance of males, which differs from some studies where most cases occurred in females [29–31]. However, many other reports clearly show a higher frequency in males [26,27,32,33]. WHO monitors gender distribution of cases of leprosy in its global and regional reports. While there is no environmental or biologic reason for a disproportionate number of cases of leprosy by gender, WHO recognizes that in certain regions or countries, women and girls are being left undiagnosed and untreated due to inequality in access to health services, education, literacy and participation in society [34]. In Cuba, health is a free, public and equity-based right encompassing a national scope and relying heavily on primary health care activities but also on specialized and personalized medical care. Dr. Carissa Etienne, director of PAHO in a report in 2018 stated: *“Cuba is one of the countries that shows the greatest progress in the implementation of the Strategy for Universal Access to Health and Universal Coverage of Health of the Pan American Health Organization (PAHO)*, *known as Universal Health*. *Cuba’s successes in health are recognized worldwide and show a consistent and systematic level of commitment to health development by the highest authorities in that country since 1959”* [35].

The province of Granma is the territory with the highest rate of detection of leprosy cases in Cuba in recent years. It also contributes with the highest number of cases in children, also maintaining the notification during almost all the years of the study, which indicates that transmission persists [13]. This result could be explained by ongoing effective surveillance activities carried out in this province for several years and by having the most experienced provincial program director in the country. However, it may also represent an indicator of the level of transmission of the infection and the risk of exposure by untreated sources of infection in this community.

The marked predominance of multibacillary cases is striking, which differs from most reports of childhood leprosy, where paucibacillary cases usually predominate [12,27,36–38]. Nevertheless, some studies report a higher proportion of multibacillary cases in the pediatric age similar to our study [39–41]. It is worth noting that these findings do not allow predicting trends; however, this indicate that children are in contact with cases of multibacillary leprosy in adults, which is the most frequent form of leprosy in adults in Cuba [17]. In this regard, Narang et al asserts that the risk can be up to 14 times higher when the contact is multibacillary, especially in lepromatous forms of the disease [42]. Additionally, this might also be explained by underlying genetic susceptibility [6].

Frequently, most reports of childhood leprosy demonstrate smear negativity at the time of the initial diagnosis [12,27,32,36,43,44]. Conversely, most of the children in our study had a positive smear microscopy at the time of diagnosis. It should be noted, however, that 34% of cases had bacillary index “one”; this technically indicates that it is observed at microscope an average of 1 to 10 bacilli in 100 fields observed [45]. This finding may be explained by the highly experienced laboratory technicians at the national reference laboratory of leprosy located at the Tropical Medicine Institute “Pedro Kourí”. This surveillance strategy could have favored the increasing diagnostic sensitivity since this facility conducts smear microscopy for the entire country of new cases and during their follow-up. Children with irreversible deformity and disability face many difficulties in education, social life, and daily activities. One of the objectives of the global leprosy strategy 2016–2020 is zero new childhood cases with grade 2 disability [23]. According to the global report by WHO in 2019, 125 countries reported cases using this indicator, of which 97 reported 0 cases and 21 countries reported between 1 and 10 cases, and only seven countries reported more than 10 cases [9].

Fortunately, almost all the children in this study did not develop neurologic disabilities like most case series of leprosy in childhood [27,29,32]. Cuba has a comprehensive nationwide healthcare system, where the NLCP follows scientific and technical guidance by the Pan-American Health Organization. As a result of leprosy control activities in Cuba, there has been substantial achievements in the fight against leprosy. To support leprosy control activities, a multidisciplinary commission of experts was established to evaluate all children suspected of leprosy referred from the provinces. The national commission consists of a pediatric neurologist, a microbiologist, an epidemiologist, and a senior dermatologist. The commission carries out evaluations at the Academic Pediatric Hospital “Juan Manuel Marquez”, in Havana City. This commission is responsible for evaluating every suspect case and confirm the diagnosis or rule it out. Children are evaluated twice by members of this commission: at the time of diagnosis and at the completion of the treatment regimen. The provincial commission carries out the follow up of these cases during treatment and after completing therapy. We consider that this strategy has improved our ability to evaluate, confirm, and manage suspected cases of childhood leprosy.

Promoting active periodic screening among contacts is essential, since the greatest source of infection in children is prolonged contact with untreated household cases and with close neighbors [29,32,41]. A child’s risk of developing leprosy if the child is exposed to a sick neighbor increases up to 4 times. If the source case is within the home, there is a nine-fold risk increase of the child acquiring the infection [42]. In our study, we identified a source case of infection or a strong family history of leprosy [12,29]. According to the last population census in 2012 in Cuba, there is a significant number of multigenerational households that could favor household transmission of leprosy due to overcrowding [46]. In this context, grandparents turned out to be the most frequently involved relative. This finding correlates with the Cuban tradition of grandparents playing an important role in childcare, particularly of children in their first years of life [47]. Other authors have suggested the special bond between mother and children when the mother is the index case [41].

The diagnosis of a single case of childhood leprosy in Cuba is as a "sentinel event" that generates an exhaustive investigation from the NLCP and the primary health care. As it was previously explained, the report of cases of childhood leprosy in Cuba is very low, and it is unlikely that family medicine physicians have the adequate exposure during their training to identify cases. The NLCP establishes that when a new case of leprosy is suspected in a primary health care area and the national commission confirms the diagnosis, the family medicine physician along with the local epidemiologist, and a dermatologist jointly institute control activities. In addition, as a part of continue education all the medical doctors who practice in the location where the case is identified, participate in the discussion of epidemiological and clinical findings, particularly in cases where the diagnosis is delayed and/or there is grade II disability. According to our results, 40% of child cases are detected through active case finding. This finding suggests that without active surveillance activities (including contact tracing and the surveillance in high-risk areas), we would have missed the opportunity to identify more than half of the cases. This shows the need for strengthening active surveillance activities and high-quality contact investigations.

The peculiarity that in our study most of the children were classified as “the only one case identified” in the foci indicate deficiencies in the epidemiological investigation at the locality level reporting the case; this is something previously reported in other Cuban studies [17].

### Limitations

This descriptive study was done conducting a retrospective data analysis of demographic, clinical, and epidemiological information from the NLCP database and therefor only include those patients who were reported. However, the results of our study provide a rough indicator of ongoing transmission of leprosy in Cuba and which can serve as a baseline parameter upon which future studies can be designed. Additionally, this information assists policymakers in Cuba to effective plan patient-oriented leprosy services and allocation of existing scarce resources.

In summary, our study demonstrates that there is ongoing transmission of leprosy in Cuba as we continue to identify new cases of childhood leprosy. Most pediatric cases are of the multibacillary type, and in most cases, there were relatives concomitantly diagnosed with leprosy or there was a next of kin with a history of treated leprosy. It appears that in Cuba, grandparents play a significant role in the transmission of leprosy during childhood. Further surveillance efforts during primary care interventions may provide the tools for the earlier identification of cases to institute interventions that may ultimately allow us to achieve the noble goal of interrupting leprosy transmission in Cuba.
